# *Sulfurimonas gotlandica* sp. nov., a chemoautotrophic and psychrotolerant epsilonproteobacterium isolated from a pelagic redoxcline, and an emended description of the genus *Sulfurimonas*

**DOI:** 10.1099/ijs.0.048827-0

**Published:** 2013-11

**Authors:** Matthias Labrenz, Jana Grote, Kerstin Mammitzsch, Henricus T. S. Boschker, Michael Laue, Günter Jost, Sabine Glaubitz, Klaus Jürgens

**Affiliations:** 1 IOW Leibniz Institute for Baltic Sea Research Warnemuende (IOW), Germany; 2 Royal Netherlands Institute of Sea Research (NIOZ), Yerseke, Netherlands; 3 Arbeitsbereich Medizinische Biologie und Elektronenmikroskopisches Zentrum (EMZ), Universität Rostock, Germany

## Abstract

A psychro- and aerotolerant bacterium was isolated from the sulfidic water of a pelagic redox zone of the central Baltic Sea. The slightly curved rod- or spiral-shaped cells were motile by one polar flagellum or two bipolar flagella. Growth was chemolithoautotrophic, with nitrate or nitrite as electron acceptor and either a variety of sulfur species of different oxidation states or hydrogen as electron donor. Although the bacterium was able to utilize organic substances such as acetate, pyruvate, peptone and yeast extract for growth, these compounds yielded considerably lower cell numbers than obtained with reduced sulfur or hydrogen; in addition, bicarbonate supplementation was necessary. The cells also had an absolute requirement for NaCl. Optimal growth occurred at 15 °C and at pH 6.6–8.0. The predominant fatty acid of this organism was 16 : 1ω7*c*, with 3-OH 14 : 0, 16 : 0, 16 : 1ω5*c*+*t* and 18 : 1ω7*c* present in smaller amounts. The DNA G+C content was 33.6 mol%. As determined in 16S rRNA gene sequence phylogeny analysis, the isolate belongs to the genus *Sulfurimonas*, within the class *Epsilonproteobacteria*, with 93.7 to 94.2 % similarity to the other species of the genus *Sulfurimonas*, *Sulfurimonas autotrophica*, *Sulfurimonas paralvinellae* and *Sulfurimonas denitrificans*. However, the distinct physiological and genotypic differences from these previously described taxa support the description of a novel species, *Sulfurimonas gotlandica* sp. nov. The type strain is GD1^T^ ( = DSM 19862^T^ = JCM 16533^T^). Our results also justify an emended description of the genus *Sulfurimonas*.

Deep-sea vents are among the most productive marine systems on Earth. The discovery of these primarily chemoautotrophic environments, in 1977, has been followed by an appreciation of the remarkable physiological and phylogenetic diversity of their endosymbiotic and often thermophilic inhabitants, most commonly species of the class *Epsilonproteobacteria*. Moreover, deep-sea vent chemolithoautotrophs are thought to be representatives of the earliest biological communities on Earth (see the review by Nakagawa & Takai, 2008). Indeed, many epsilonproteobacteria are globally ubiquitous in oxygen-deficient and sulfide-rich marine and terrestrial ecosystems, which accommodate their predominantly auto- to mixotrophic lifestyles (Campbell *et al.*, 2006). A number of studies have verified the significant role of epsilonproteobacteria in biogeochemical cycles, particularly those which are sulfur-dependent, as is the case in deep-sea hydrothermal fields (Nakagawa *et al.*, 2005; Campbell *et al.*, 2006), sulfidic cave springs (Engel *et al.*, 2004) and autotrophic episymbiotic associations (Suzuki *et al.*, 2006). In the suboxic to sulfidic transition zones of aquatic pelagic redox zones, high dark CO_2_ fixation rates, mainly due to the activities of epsilonproteobacterial chemolithoautotrophs, have been determined, for instance, in the Black Sea and the Baltic Sea (Grote *et al.*, 2008; Glaubitz *et al.*, 2010; Jost *et al.*, 2008).

The Baltic Sea is among the largest brackish basins of the world, with periodically anoxic conditions in its bottom waters. In the region known as the Baltic Proper there are a number of such areas, including the Gotland Deep, where at depths below 50–60 m a stable halocline separates the water column into an upper oxygenated layer and underlying oxygen-deficient and anoxic/sulfidic layers (Lepland & Stevens, 1998; Neretin *et al.*, 2003), in which high dark CO_2_ fixation rates have been reported (Jost *et al.*, 2010).

In stimulation experiments (Labrenz *et al.*, 2005; Brettar *et al.*, 2006), quantitative 16S rRNA PCR (Labrenz *et al.*, 2004), catalysed reporter deposition–fluorescence *in situ* hybridization (CARD-FISH; Grote *et al.*, 2007) and microautoradiography (MICRO)-CARD-FISH (Grote *et al.*, 2008) analyses, as well as 16S rRNA stable isotope probing (RNA-SIP; Glaubitz *et al.*, 2009), the epsilonproteobacterial ‘Uncultured *Helicobacteraceae* G138eps1/GD17’ subgroup was shown to account for up to 30 % of the total cell numbers in pelagic redox zones of the central Baltic Sea. The abundance of these bacteria highlights the importance of chemolithoautotrophic denitrification, which was convincingly demonstrated to be the major N-loss process in water columns with a sulfide–nitrate interface (Brettar & Rheinheimer, 1991; Hannig *et al.*, 2007; Jensen *et al.*, 2009), catalysed by the GD17 group as potential key organisms for this process. According to its 16S rRNA phylogeny, the ‘Uncultured *Helicobacteraceae* G138eps1/GD17’ subgroup belongs to the genus *Sulfurimonas**,* which comprises mesophilic, facultatively anaerobic, chemolithoautotrophic species originating from deep-sea hydrothermal and marine sulfidic environments (Takai *et al.*, 2006). In previous work (Grote *et al.*, 2012) we described the isolation of strain Gotland Deep 1 (GD1^T^), a close phylogenetic relative (16S rRNA similarity of 95.7 %) and thus representative of the Baltic *Sulfurimonas* ‘Uncultured *Helicobacteraceae* G138eps1/GD17’ subgroup. Selected genomic and physiological data suggested an ecological role for GD1^T^, especially with respect to its sulfide detoxification ability (Grote *et al.*, 2012). Here, we expand on previous work by presenting the taxonomic characteristics of GD1^T^. Our results form the basis of an emended description of the genus *Sulfurimonas*.

Strain GD1^T^ was isolated from a pelagic redox zone of the Gotland Deep in the central Baltic Sea during a research cruise on board the RV *Alkor* in May 2005 (57° 19.2′ N 20° 03′ E). Water was collected in a free-flow bottle attached to a CTD-rosette from a depth of 215 m. The *in situ* temperature was 6 °C, the salinity 13 practical salinity units (PSU), and the sulfide concentration 11 µM. Directly on board, 100 µM KNO_3_ and 100 µM Na_2_S_2_O_3_ were added to the water samples, which were then incubated in the dark at 10 °C under anoxic conditions. For further isolation and cultivation in the laboratory, a modified version of artificial brackish water medium (ABW) (Bruns *et al.*, 2002) was used, consisting of 95 mM NaCl, 11.2 mM MgCl_2_ . 6H_2_O, 2.3 mM CaCl_2_ . 2H_2_O, 2.0 mM KCl, 6.4 mM Na_2_SO_4_, 192 µM KBr, 92 µM H_3_BO_3_, 34 µM SrCl_2_, 92 µM NH_4_Cl, 9 µM KH_2_PO_4_ and 16 µM NaF, buffered with 10 mM HEPES (pH 7.3). For anaerobic cultivation, the medium was boiled, bubbled with N_2_ for 30 min, and then autoclaved under anoxic conditions. Subsequently, anoxic and sterile-filtered 0.1 % (v/v) of the trace element solution SL10 (Widdel *et al.*, 1983), 0.2 % (v/v) of a 10-vitamin solution (Balch *et al.*, 1979), 0.02 % (v/v) of a selenite–tungstate solution (Widdel & Bak, 1992), and 2–5 mM NaHCO_3_ were added. The standard medium ABW+nitrate+thiosulfate (ABW+NS) was prepared by the variable addition of 10 mM KNO_3_ and 10 mM Na_2_S_2_O_3_, with the final concentration depending on the experiment. A pure culture was acquired by the dilution to extinction method and was cryopreserved at −80 °C in glycerol for long-term storage.

Morphological, physiological, and metabolic characteristics were, for the most part, analysed as described earlier (Grote *et al.*, 2012). For these analyses, strain GD1^T^ was cultivated in triplicate for 7–10 days at 15 °C in the dark. Growth was usually measured by counting 4′,6′-diamidino-2-phenylindol (DAPI) stained cells, observed using epifluorescence microscopy, or by flow cytometric determinations of SYBR-Green I (Molecular Probes) stained cells (Labrenz *et al.*, 2007) at the end of the experiment. *Sulfurimonas denitrificans* DSM 1251^T^ was used as the reference strain in the cultivation experiments.

Isolate GD1^T^ is a motile, Gram-reaction-negative, slightly curved or spirilla-shaped bacterium typically with one polar flagellum (Fig. 1a, b), but in some cases two flagella at opposite poles (Fig. 1c). Cell width was rather constant (mean = 0.66 µm, sd = 0.083 µm, *n* = 112) whereas cell length, i.e. from pole to pole, was variable (mean = 2.1 µm, sd = 0.54 µm, *n* = 112). The cells had a positive chemotactic response to nitrate (Grote *et al.*, 2012). Under optimal conditions in ABW+NS medium the cell doubling time of strain GD1^T^ was 13 h. Cells in older cultures tended to form aggregates. Growth at temperatures in the range of 4–40 °C was investigated, with highest cell numbers obtained between 4 and 20 °C and optimal growth at 15 °C (Grote *et al.*, 2012). Thus, isolate GD1^T^ is the first psychrotolerant species within the genus *Sulfurimonas*, in which all member species at the time of writing are mesophilic (Table 1).

**Fig. 1.**
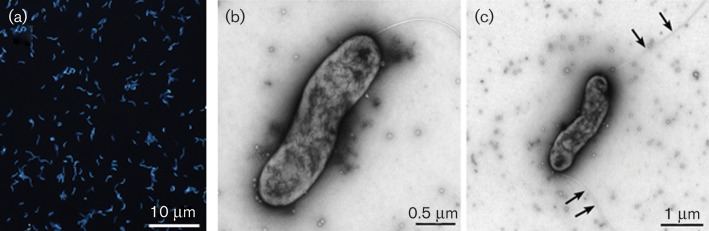
Cell morphology of spirilla-shaped cells of strain GD1^T^ cultivated on ABW+NS medium. (a) Fluorescence microscopy of 4′,6′-diamidino-2-phenylindol (DAPI) stained cells. (b) Transmission electron microscopy of a bacterium with one flagellum and (c) of a bacterium with two flagella (indicated by arrows), both negatively stained with phosphotungstic acid.

**Table 1.** Differential characteristics between strain GD1^T^ and species of the genus *Sulfurimonas* Taxa: 1, *Sulfurimonas gotlandica* sp. nov. GD1^T^; 2, *Sulfurimonas denitrificans* DSM 1251^T^ (data from this study; Timmer-ten Hoor, 1975; Brinkhoff *et al.*, 2005); 3, *Sulfurimonas paralvinellae* GO25^T^ (Takai *et al.*, 2006); 4, *Sulfurimonas autotrophica* OK10^T^ (Inagaki *et al.*, 2003). nd, Not determined; +, positive; −, negative.

Characteristic	1	2	3	4
Morphology				
Cell shape	Curved rods to spirilla-like	Rods to spirilla-like	Rods	Rods
Motility	+	−	+	+
Growth				
Anaerobic growth	+	+	+	-
Doubling time under optimal conditions (h)	13	12	13–16	1.4
Temperature dependence	Psychrotolerant	Mesophilic	Mesophilic	Mesophilic
Temperature range (°C)	4–20	10–30	4–35	10–40
Temperature optimum (°C)	15	22	30	23–26
pH range	6.5–8.4	nd	5.4–8.6	5.0–9.0
pH optimum	6.7–8.0	7	6.1	6.5
NaCl requirement	+	−	+	+
Maximum O_2_ concentration (%)	10–<20	0.5	10	15
Inorganic electron donors	H_2_, HS^−^, S^0^, S_2_O_3_^2-^	HS^−^, S_2_O_3_^2-^	H_2_, S^0^, S_2_O_3_^2-^	S^0^, S_2_O_3_^2-^
Organic electron donors	Formate, acetate, yeast extract, pyruvate, amino acid mix	Formate, fumarate, yeast extract, alcohol mix	−	−*
Electron acceptors	NO_3_^−^, NO_2_^−^	NO_3_^−^, NO_2_^−^, O_2_	NO_3_^−^, O_2_	O_2_
Fatty acids (mol%)				
14 : 0	0.9	0.4	5	8.4
3-OH 14 : 0	2.5		7	
16 : 1ω7*c*	66.0	67.9	22†	45.2†‡
16 : 1ω5*c*+*t*	1.3	2.0		
16 : 0	15.5	15.3	25	37.1
18 : 0			4	
18 : 1ω7*c*	13.1	12.1	37†	
18 : 1*trans*				9.4§
DNA G+C content (mol%)	33.6‖	36	37.6	35.2

* Tested without bicarbonate supplementation.

† No differentiation *in cis*/*trans*.

‡ Identified as 16 : 1*cis.*

§ Potentially also 18 : 1ω7*c*, which has a similar retention time to 18 : 1ω9*t*.

‖ Based on genome analyses.

To obtain media with different pH values, the pH of a 20 ml subsample from the anoxic ABW+NS was adjusted to pH 6.0, 6.5, 6.7, 6.9, 7.1, 7.5, 8.0, 8.4 and 9.0 by the addition of the appropriate amount of 0.1M HCl. For the experimental setup, the corresponding amount of 1 M HCl was added to the media preparations, which were then inoculated. After 14 days of incubation, the pH was measured. At an initial pH of 6.5–8.4, it remained constant (±0.02) throughout the experiment whereas below and above this range it decreased by about 0.18–0.25 pH units. Optimal growth occurred over a wide pH range (6.7–8.0) but no growth occured at pH 6.0 and 8.4. The NaCl requirement was determined by cultivation in ABW+NS containing the following salt concentrations [NaCl (g l^−1^)/MgCl_2_ . 6H_2_O (g l^−1^)]: 0/0, 0/0.50, 2.50/0.38, 5.00/0.75, 7.50/1.13, 10.00/1.50, 12.50/1.88, 15.00/2.25, 17.50/2.63 and 20.00/3.00. The isolate had an absolute requirement for NaCl and grew best with between 10 and 20 g NaCl l^−1^; the upper limit for growth was not further determined. No growth was observed in media without added NaCl, in contrast to *Sulfurimonas denitrificans* DSM 1251^T^, which grew equally well without NaCl and at all NaCl concentrations tested (Table 1).

To identify the electron donors sustaining chemoautotrophic growth of isolate GD1^T^, ABW medium containing 5 mM nitrate was supplemented with sulfite (1 mM), sulfide (10 µM, 20 µM, 100 µM) or elemental sulfur (1 mM). Hydrogen utilization was assessed by bubbling ABW+NS with forming gas (N_2_/H_2_, 95 : 5) for several hours prior to inoculation and cultivation. Strain GD1^T^ was able to use all of the tested electron donors as an energy source for growth although growth was inhibited by sulfide concentrations >20 µM (Grote *et al.*, 2012). This observation is in accordance with *in situ* activities of chemoautotrophic micro-organisms in pelagic Gotland Deep redox zones, where dark CO_2_ fixation rates are significantly reduced at environmental sulfide concentrations >20 µM (Jost *et al.*, 2010). As electron acceptors, nitrate (100 µM, 2 mM, 5 mM, 10 mM), nitrite (600 µM, 2 mM) (Grote *et al.*, 2012), manganese(IV) oxide (200 µM), manganese(III) acetate dihydrate (2.4 mM), iron(III) chloride hexahydrate (5 mM), fumarate (100 µM) and oxygen (4 % saturation, approx. 12 µmol O_2_ l^−1^) were tested in ABW containing 5 mM thiosulfate. For the oxygen experiment, the oxygen content in fully oxygenated ABW+thiosulfate was measured with an optode (POF-PSt3; PreSens) and the appropriate amount of oxygen was then mixed with anoxic ABW+thiosulfate to achieve the desired amount of saturation. However, only nitrate and nitrite served as electron acceptors during growth of the bacterium.

Although the manganese and iron concentrations tested may have been too high and thereby suppressed cell growth, previous thiosulfate/manganese stimulation experiments with Baltic Sea water samples containing lower metal concentrations similarly failed to reveal active manganese-reducing species of the genus *Sulfurimonas* (Labrenz *et al.*, 2005). *Sulfurimonas autotrophica* is likewise unable to reduce ferrihydrite (Inagaki *et al.*, 2003), which further supports the lack of direct participation of strain GD1^T^ in the Mn/Fe-shuttle (Neretin *et al.*, 2003) of Baltic pelagic redox zones. It also cannot be excluded that strain GD1^T^ is able to grow in medium with an oxygen concentration below 4 %, given that the genome of this bacterium includes a gene encoding a putative cbb3-type cytochrome *c* oxidase with the potential to mediate aerobic respiration (Grote *et al.*, 2012). If aerobic respiration could occur at very low oxygen concentrations, it was beyond the scope of our experimental design. The oxygen sensitivity of strain GD1^T^ was examined in detail, using ABW+NS with oxygen saturations of 0.5, 3, 5, 10, 20, 30, 40 and 50 %. Compared to oxygen-free conditions, oxygen concentrations ≥20 % reduced or inhibited the growth of this strain whereas oxygen concentration ≤10 % had no such effect (Grote *et al.*, 2012). Thus, the oxygen tolerance of strain GD1^T^ is similar to that of aerobic *Sulfurimonas autotrophica* OK10^T^ (Table 1). Based on our current knowledge, we consider strain GD1^T^ to be an aerotolerant representative of the genus *Sulfurimonas*.

Chemolithoautotrophic growth was directly confirmed in ABW+NS containing ^14^C-bicarbonate followed by a combination of fluorescence *in situ* hybridization and microautoradiography (MICRO-CARD-FISH) (Grote *et al.*, 2012). As electron donor (in ABW+5 mM KNO_3_) alone or as electron donor and sole carbon source (in NaHCO_3_-free ABW+5 mM KNO_3_) the following compounds were tested: (a) glucose (0.1 mM), (b) a mixture of lactate, malate, fumarate, succinate, glycerine and glucose (abbreviated as mix 4) (100 µM), (c) yeast extract (0.01 mg l^−1^), (d) pyruvate (100 µM), (e) acetate (100 µM), (f) fumarate (100 µM), (g) alcohol mix (butanol, ethanol, methanol, propanol; 100 µM) (Grote *et al.*, 2012) and (h) an amino acid mix (0.1 mM) consisting of (g l^−1^): β-alanine 0.466, l-arginine 0.872, l-asparagine 0.750, l-cysteine 0.606, l-glutamine 0.730, l-glutamic acid 0.736, glycine 0.376, isoleucine 0.656, l-leucine 0.656, l-methionine 0.746, l-phenylalanine 0.826, l-serine 0.526, l-threonine 0.596, l-valine 0.586, l-proline 0.576, l-tryptophan 1.022, l-histidine 0.776, l-lysine 0.822, l-tyrosine 0.906 and l-asparagine 0.666.

In the presence of 2 mM NaHCO_3_, the growth of isolate GD1^T^ was promoted with formate, acetate, yeast extract, pyruvate and the amino acid mix as electron donors. However, maximal cell numbers were usually more than a magnitude less than those reached with thiosulfate/nitrate-containing medium, as shown in Fig. 2(a) for pyruvate, which was also used in radiotracer experiments aimed at confirming the capability of strain GD1^T^ to use organics as electron donor. In those experiments, CO_2_ production was measured following the addition of 16 kBq [2-^14^C]pyruvate (specific activity 0.6 GBq mmol^−1^) to cultures grown solely on pyruvate or on thiosulfate/pyruvate. After 24 h or 72 h of incubation, CO_2_ was degassed by the acidification of cell-free medium and trapped in ethanolamine. In nitrate/pyruvate medium, the growth of strain GD1^T^ was accompanied by elevated CO_2_ production (Fig. 2b). The simultaneous incorporation of [2-^14^C]pyruvate into GD1T cells was much less pronounced, but its uptake and contribution to biomass production were clearly determined in thiosulfate/nitrate/pyruvate medium, where total cell numbers were also higher than those reached in thiosulfate/nitrate medium (Fig. 2a), but the difference was not statistically significant (unpublished data). By contrast, in NaHCO_3_-free medium strain GD1^T^ was unable to use any of the organics offered simultaneously as electron donor and carbon source (Fig. 2a). It has long been recognized that even heterotrophic bacteria may require CO_2_ for growth (Dehority, 1971), e.g. in anaplerotic reactions (Alonso-Sáez *et al.*, 2010). Similar findings were reported for *Nitrobacter hamburgensis*, which requires atmospheric CO_2_ or the addition of sodium carbonate for mixotrophic growth (in the presence of NO_2_^−^) on d-lactate (Starkenburg *et al.*, 2008). The authors of that study suggested that CO_2_ fixation served as a reductant sink necessary to maintain cellular redox balance. The physiological background for the growth of isolate GD1^T^ on organics is thus far unclear. In other species of the genus *Sulfurimonas*, organic substance utilization is variable. For example, in a similar experiment *Sulfurimonas denitrificans* was able to use formate, fumarate, yeast extract and the alcohol mix as electron donors (Table 1). The ability of this bacterium to oxidize formate was proposed in a genome analysis, which identified a formate dehydrogenase complex (Sievert *et al.*, 2008). Homologues of genes involved in glycolysis and proteolysis are also present in the genome of strain GD1^T^ (Grote *et al.*, 2012), whereas *Sulfurimonas autotrophica* (Inagaki *et al.*, 2003; but tested without bicarbonate supplementation to the organic medium) and *Sulfurimonas paralvinellae* (Takai *et al.*, 2006) are unable to grow on organic compounds. In conclusion, although under specific circumstances organic compounds enhance the growth of some species of the genus *Sulfurimonas*, members of this genus characteristically grow chemolithoautotrophically.

**Fig. 2.**
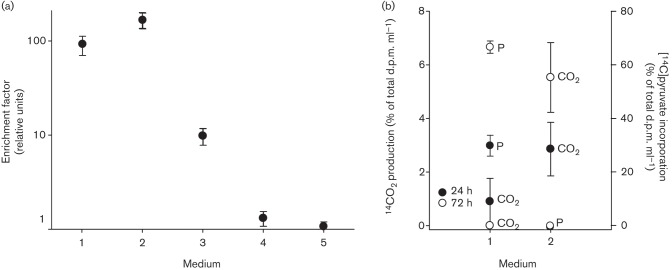
Impact of pyruvate on the growth of isolate GD1^T^. Error bars indicate the standard deviation of three independent replicates for each assay. (a) Growth on media with different substrate combinations: 1, NaHCO_3_, S_2_O_3_^2-^, NO_3_^−^; 2, NaHCO_3_, S_2_O_3_^2-^, NO_3_^−^, pyruvate; 3, NaHCO_3_, pyruvate; 4, pyruvate; 5, ABW without further supplements. The relative enrichment factor describes the increase of cell numbers after 7 days of incubation compared to the initial cell numbers after inoculation at day 0 (6.1×10^5^ ml^−1^). (b) ^14^CO_2_ production and [^14^C]pyruvate incorporation after 24 h and 72 h of incubation. Media: 1, NaHCO_3_, S_2_O_3_^2-^, NO_3_^−^, [^14^C]pyruvate; 2, NaHCO_3_, NO_3_^−^, [^14^C]pyruvate. P, pyruvate incorporation; CO_2_, CO_2_ production.

Total fatty acids and phospholipid-derived fatty acids were extracted as described by Sasser (1990) and Boschker (2004), respectively, and analysed by gas chromatography with a flame-ionization detector on a non-polar HP-5ms column (Agilent). The dominant cellular fatty acid of strain GD1^T^ was 16 : 1ω7*c*, with 3-OH 14 : 0, 16 : 0, 16 : 1ω5*c*+*t*, and 18 : 1ω7*c* detected in lower amounts. This fatty acid profile is comparable to those of other species of the genus *Sulfurimonas* but most similar to that of *Sulfurimonas denitrificans* (Table 1). This may reflect the fact that strain GD1^T^ and *Sulfurimonas denitrificans* were cultivated on ABW+NS under identical conditions. However, a high percentage of C16 : 0 and one or both of the monounsaturated C16 and C18 fatty acids has also been described in other members of the class *Epsilonproteobacteria*, such as *Nitratifractor salsuginis* and *Sulfurovum lithotrophicum* (Suzuki *et al.*, 2005). Accordingly, this combination may be a general characteristic of these epsilonproteobacteria.

The DNA guanine-plus-cytosine (G+C) content of strain GD1^T^ was determined to be 33.6 mol%, as calculated by analysis of the whole genome (Grote *et al.*, 2012).

To establish the closest relatives of strain GD1^T^ based on 16S rRNA sequencing, preliminary searches in the EMBL Data Library were performed with the program fasta (Pearson & Lipman, 1988). Closely related sequences were retrieved from GenBank and aligned and analysed with the newly determined sequence, within the program arb (Ludwig *et al.*, 2004). Sequences for analysis were reduced to unambiguously alignable positions using group-specific filters. For phylogenetic analyses, three different trees were calculated using the neighbour-joining, parsimony and maximum-likelihood (Phyml) algorithms based on nearly full-length 16S rRNA sequences (approx. 1400 bp). For neighbour-joining, the Jukes–Cantor-correction was applied. Shorter sequences were gradually inserted into the reconstructed tree without changing the topology. Sequence searches of the EMBL database (latest: 2013-05-14) revealed that our isolate is related to the epsilon class of the phylum *Proteobacteria* (data not shown). In a pairwise analysis, it displayed highest (93.7–94.2 %) 16S rRNA gene sequence similarity to species of the genus *Sulfurimonas* and to the Baltic ‘Uncultured *Helicobacteraceae* G138eps1/GD17’ subgroup (95.7 %). Lower levels of relatedness (≤91 % sequence similarity) were determined for the other examined species belonging to the epsilon class of the phylum *Proteobacteria*.

An unrooted tree reconstructed using the neighbour-joining method showed the phylogenetic position of the novel bacterium, strain GD1^T^, amongst the members of the class *Epsilonproteobacteria* (Fig. 3). Treeing analyses confirmed it to be a member of the genus *Sulfurimonas*, forming a stable cluster with the ‘Uncultured *Helicobacteraceae* G138eps1/GD17’ subgroup. This cluster is specifically detected by the SUL90 16S rRNA gene probe, originally developed to be 100 % complementary to the G138eps1/GD17 target site (Grote *et al.*, 2007).

**Fig. 3.**
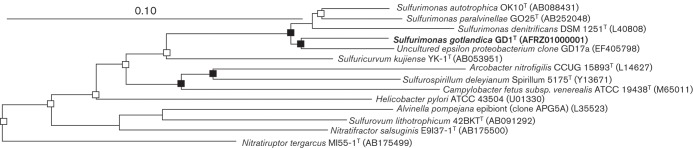
Unrooted tree showing phylogenetic relationships of isolate GD1^T^ and closely related members of the class *Epsilonproteobacteria*. The tree was reconstructed using the neighbour-joining method and was based on a comparison of approximately 1400 nt. Solid squares indicate that the corresponding nodes (or groups) were recovered in neighbour-joining, maximum-parsimony and maximum-likelihood methods. Branching points supported by two algorithms are marked by an open square. The following strains were used as an outgroup (not shown): *Antarctobacter heliothermus* EL-219^T^, *Sagittula stellata* E-37^T^, *Roseovarius tolerans* EL-172^T^, *Roseovarius nubinhibens* ISM^T^ and *Roseovarius mucosus* DFL-24^T^. Bar, 1 substitution per 10 nt.

There is no precise correlation between percentage 16S rRNA sequence divergence and species delineation, but it is generally recognized that divergence values ≥3 % are significant (Stackebrandt & Goebel, 1994). However, it is pertinent to note that the phylogenetic separateness of strain GD1^T^ is strongly supported by phenotypic considerations. For instance, this novel bacterium is distinguishable from other species of the genus *Sulfurimonas* by its psychrotolerance and energy metabolism (Table 1). Additional characteristics useful in differentiating Baltic isolate GD1^T^ from related organisms are shown in Table 1. Based on phenotypic and genetic evidence, we propose the classification of strain GD1^T^ as a representative of a novel species of the genus *Sulfurimonas*: *Sulfurimonas gotlandica* sp. nov.

## Emended description of the genus *Sulfurimonas*

The description is based on that by Takai *et al.* (2006). Cells are Gram-negative and morphologically variable. Straight to slightly short rods, elongated rods and spiral in different growth phases and under different growth conditions. Psychrotolerant to mesophilic and aerotolerant to facultatively anaerobic. Do not always require NaCl for growth. Optimal growth occurs chemolithoautotrophically with sulfide, S^0^, thiosulfate and H_2_ as electron donors, and with nitrate, nitrite and O_2_ as electron acceptors, using CO_2_ as a carbon source. Supplementation of bicarbonate can enable growth on organic substances, but yields much lower cell numbers compared to growth on reduced sulfur or hydrogen. Potential ecological niches are deep-sea hydrothermal environments and benthic or pelagic marine to brackish transition zones from oxic to anoxic/sulfidic environments. The type species is *Sulfurimonas autotrophica* (Inagaki *et al.* 2003).

## Description of *Sulfurimonas gotlandica* sp. nov.

*Sulfurimonas* *gotlandica* (got.lan′di.ca. N.L. fem. adj. *gotlandica* pertaining to the Gotland Deep, the basin in the central Baltic Sea from which the organism was first isolated).

Gram-negative, slightly curved or spirilla-shaped cells. Motile by one polar flagellum or two flagella at opposite poles. Cells exhibit a positive chemotactic response to nitrate. Cell sizes are 0.66±0.083×2.1±0.54 µm. Cells have a tendency to aggregate at older stages. Psychro- and aerotolerant. The temperature range for growth is 4–20 °C. Optimal growth occurs at 15 °C and pH 6.7–8.0. The cells have an absolute requirement for NaCl. Chemolithoautotrophic growth occurs with H_2_, HS^−^, S^0^ and thiosulfate. Supplementation of bicarbonate can enable growth on formate, acetate, yeast extract, pyruvate or amino acid mix, but yields much lower cell numbers compared with growth on reduced sulfur or hydrogen. Sulfide concentrations of more than 20 µM inhibit, but up to 10 % of oxygen in the medium does not influence growth. Dominant cellular fatty acid is 16 : 1ω7*c*, with 14 : 0, 16 : 0, 16 : 1ω5*c*+*t*, and 18 : 1ω7*c* present in smaller amounts.

The type strain is GD1^T^ ( = DSM 19862^T^ = JCM 16533^T^), isolated from water of a pelagic redox zone of the central Baltic Sea. The G+C content of the type strain is 33.6 mol%.
